# Ultrasound-assessed perirenal fat is related to increased ophthalmic artery resistance index in HIV-1 patients

**DOI:** 10.1186/1476-7120-8-24

**Published:** 2010-06-30

**Authors:** Pierfrancesco Grima, Marcello Guido, Roberto Chiavaroli, Antonella Zizza

**Affiliations:** 1 Division of Infectious Diseases, HIV Center, S. Caterina Novella Hospital, Galatina, Italy; 2 Laboratory of Hygiene, Department of Biological and Environmental Sciences and Technologies, Di.S.Te.B.A., Faculty of Sciences, University of Salento, Lecce, Italy; 3 Institute of Clinical Physiology, National Research Council, Lecce, Italy

## Abstract

**Background:**

The introduction of highly active antiretroviral therapy (HAART) has dramatically changed the prognosis of human immunodeficiency virus (HIV) infection, with a significant decline in morbidity and mortality.

Changes in body fat distribution are a common finding in individuals with HIV infection being treated with antiretrovirals, and this condition (collectively termed lipodystrophy syndrome) is associated with depletion of subcutaneous fat, increased triglycerides and insulin resistance. Obesity, particularly visceral obesity, is associated with increased risk of cardiovascular disease. Therefore, estimating visceral fat distribution is important in identifying subjects at high risk for cardiovascular disease.

The aim of our study was to evaluate whether perirenal fat thickness (PRFT), a parameter of central obesity, is related to ophthalmic artery resistance index (OARI), an index of occlusive carotid artery disease in HIV-1 infected patients.

**Methods:**

We enrolled 88 consecutive HIV-1-infected patients receiving highly active antiretroviral therapy for more than 12 months, in a prospective cohort study. Echographically measured PRFT and OARI, as well as serum metabolic parameters, were evaluated. PRFT and OARI were measured by 3.75 MHz convex and 7.5 MHz linear probe, respectively.

**Results:**

The means of PRFT and OARI in HIV-1-infected patients with visceral obesity was considerably higher than in patients without it (p < 0.0001 and p < 0.001, respectively). Using the average OARI as the dependent variable, total serum cholesterol level, HDL, triglycerides, glycemia, sex, blood pressure, age and PRFT were independent factors associated with OARI. A PRFT of 6.1 mm was the most discriminatory value for predicting an OARI > 0.74 (sensitivity 78.9%, specificity 82.8%).

**Conclusions:**

Our data indicate that ultrasound assessment of PRFT may have potential as a marker of increased endothelial damage with specific involvement of the ocular vascular region in HIV-1-infected patients.

## Background

The introduction of highly active antiretroviral therapy (HAART) has dramatically changed the prognosis of human immunodeficiency virus (HIV) infection, with a significant decline in morbidity and mortality [1]. Treatment with antiretroviral agents has uncovered a syndrome of abnormal fat redistribution, impaired glucose metabolism, insulin resistance and dyslipidemia, collectively termed lipodystrophy syndrome [2-4] potentially contributing to cardiovascular risk [5,6]. Measurement of the inferior part of the perirenal fat has been shown to be an easy and reliable imaging indicator of visceral obesity and cardiovascular risk factors [7]. Increased cardiovascular risk has been shown to be related to carotid intima medial thickness as well as to subclinical atherosclerotic vessel damage [8-10]. Moreover, a direct correlation between intima-media thickness (IMT) of common carotid and ophthalmic artery resistance index (OARI) has been observed in HIV-1 patients [11]. Our hypothesis was that ultrasound OARI measurement may be a potential marker of increased endothelial damage as an early predictor of IMT increase.

It is important to determine which group of HIV-1 patients is at increased risk for atherosclerosis. Identification of predictive factors for the development of cardiovascular complications would be very useful, because preventive intervention could then be undertaken for HIV-1 patients.

Visceral obesity, more than overall obesity, plays a key role in the development of cardiovascular disease [12]. Thus, estimating visceral fat distribution is important in order to identify subjects with a high risk for cardiovascular disease.

Visceral obesity can be evaluated using several techniques, including ultrasound, CT (gold standard) and MRI. Measurement of visceral fat volume using ultrasound can as effective as using CT. The advantages of this method are its low cost, lack of side effects, and technical suitability [13].

It is known that there is good correlation between intra-abdominal fat distribution and ultrasonographic measurement of fat thickness and between perirenal fat thickness (PRFT) and central obesity [14-18].

We performed a prospective cohort study to identify the role of visceral fat distribution in endothelial damage and risk. The objective of our study was to evaluate whether ultrasound parameters of visceral obesity could be associated with ophthalmic blood flow, playing a role as a possible surrogate marker of cardiovascular risk in HIV-1-infected patients.

## Methods

This study was approved by the local institutional Ethics Committee and all patients approached for the study gave written consent to participate.

### Study population

HIV-1-infected patients of Santa Caterina Novella Hospital (Galatina, Italy) were considered for this study. All patients had documented HIV-1 infection, had been receiving HAART for 12 months, and were older than 18 years of age. An in-depth assessment was performed, including HIV disease history, other co-morbid conditions, medication exposure and measurement of systolic blood pressure (determined using a sphygmomanometer with the subjects at rest in a sitting position). Smokers were defined as individuals smoking more than 5 cigarettes/day at least during the past year (in our cohort all the patients who smoked declared they smoked > 5 cigarettes/day). Subjects were excluded from participating if they had any of the following clinical conditions: active AIDS-defining illness, diabetes mellitus or current use of oral hypoglycemic agents, family history of myocardial infarction (prior to age 55 for first-degree male relatives and prior to age 65 for first-degree female relatives), a history of coronary heart disease or stroke, uncontrolled hypertension, active drug abuse, alcohol abuse (defined as alcohol consumption > 30 gr/day), untreated hypothyroidism. Patients requiring systemic chemotherapy, radiation therapy or systemic steroids were excluded. Two groups of patients were consecutively selected. The first group comprised HIV-1-infected patients with a diagnosis of visceral obesity. The second group included HIV-1-infected patients for whom the diagnosis of visceral obesity had been excluded. Diagnoses of visceral obesity were based on ultrasound measured PRFD/BMI ratio > 0.22. Grima et al. [16] observed that this value could be considered a potential parameter for assessing visceral fat accumulation in HIV-1 infected patients.

### General metabolic assessment

CD4+ cell counts, HIV ribonucleic acid (RNA) load, total serum cholesterol level, high-density lipoprotein (HDL) cholesterol level, glycemia, and triglyceride levels were evaluated at baseline after a 12-h overnight fast.

**Figure 1:**
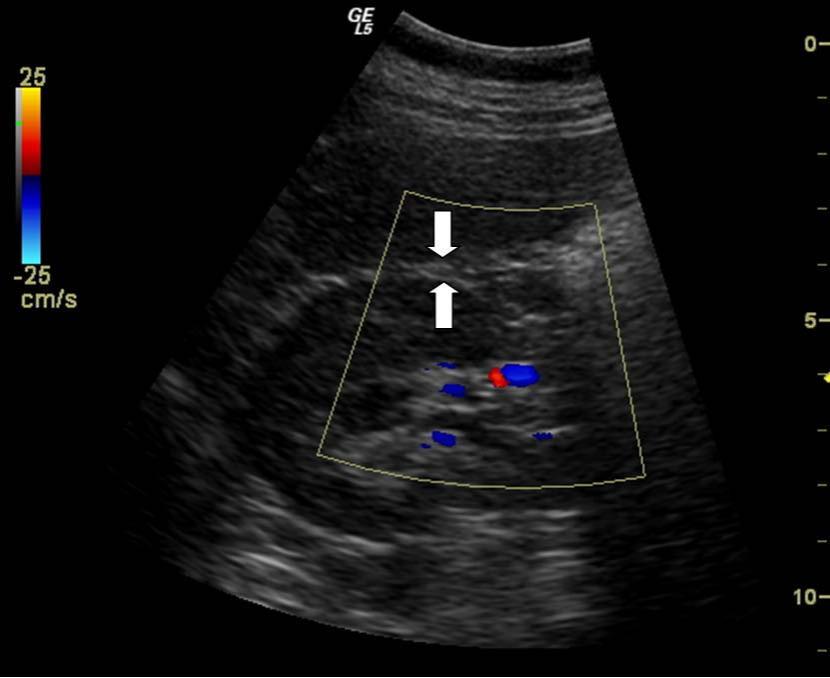
**Assessment of perirenal fat diameter**. Longitudinal scan (3.75 mHz) along the right midclavicular line, from the border of the right liver lobe to the border of the inferior pole of the right kidney. Arrows, limits of perirenal fat thickness.

**Figure 2:**
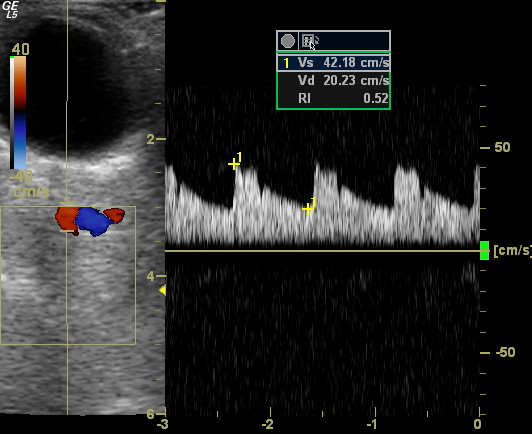
**Assessment of ophthalmic artery resistance index with patient lying supine with eyes closed and using an ultrasound frequency of 7.5 mHz**. The transducer is applied to the closed upper eyelid using a thick layer of acoustic gel, minimizing pressure on the globe.

For ultrasound measurement of visceral fat and ophthalmic artery resistance index a Logiq 5 ultrasound scanner (General Electric Medical Systems, Wallingford, Connecticut, USA) equipped with a 3.75 MHz convex and 7.5 MHz linear probe was used. Sonographic evaluation of visceral obesity was always performed by a single trained sonographer blinded to the patients' data. The visceral fat thickness was determined with a 3.75-MHz convex transducer at a specific referee point as PRFT (longitudinal scan along the right mid-clavicular line, from the border of the right liver lobe to the border of the inferior pole of the right kidney, Fig. 1) with the patient in the supine position. OARI was measured with the patient lying supine with eyes closed, using an ultrasound frequency of 7.5 mHz and by averaging the readings from at least three consecutive waveforms. The average of the values was defined as the OARI (Ophthalmic Artery Resistance Index). The transducer was applied to the closed upper eyelid using a thick layer of acoustic gel, minimizing the pressure on the globe (Fig. 2). The reproducibility of PRFT and OARI measurement was evaluated by triple determinations in 15 subjects other than the enrolled patients. A clinically significant increase in OARI was defined as a value > 0.75 according to previous observations [19].

### Statistical analysis

Continuous variables were reported as the mean ± standard deviation (SD) and categorical factors were reported as percentages. Demographic characteristics and metabolic variables were compared between the patient groups by analysis of variance (ANOVA) of successive measurements. Multiple regression analysis was used to assess the independent association between the OARI and PRFT adjusting for various risk factors. Total serum cholesterol level, HDL, triglycerides, glycemia, age, sex and blood pressure were considered as independent variables in the multivariate model.

The most discriminant cutoffs were calculated by receiver operating characteristic (ROC) curves. Statistical calculations were performed with MedCalc software, version 9.6.0.0. A p-value < 0.05 was considered to be statistically significant.

## Results

Study population. Eighty-eight patients were recruited during 2009. A total of 38 patients (25 men and 13 women) were in the visceral obesity group and 50 (33 men and 17 women) were in the control group. All subjects were of the Caucasian race. Table 1 shows patients' demographic and clinical characteristics. There were no differences between the two groups with regard to age, gender ratio, smoking status, risk factors, metabolic parameters, BMI, CD4 cell count, viral load and HAART duration. Plasma triglyceride levels were higher (although without statistical significance) in patients with visceral obesity. All HIV-1-infected patients had a backbone of Tenofovir/Emtricitabine. Protease inhibitors (PI) had been prescribed in 47 (53.4%) patients (27 with visceral obesity and 20 without obesity), without statistically significant difference between the two groups. All patients exposed to PIs received a ritonavir-boosted PI. Non-nucleoside reverse-transcriptase inhibitor (NNRTI) had been prescribed in 41 (46.5%) patients (23 with visceral obesity and 18 without obesity). The sonographic assessment of PRFT and OARI provided good reproducibility, and the intraoperator coefficient of variation was 6.2% and 8.6%, respectively.

**Table 1:** Demographic and clinical characteristics of HIV-1 patients receiving HAART

Characteristic	A (n = 88)	B (n = 38)	C (n = 50)	p-value
Age (years)	42 ± 8.6	41.4 ± 7.0	44.2 ± 8.2	NS
Sex (M/F)	42/26	33/17	25/13	NS
BMI (kg/m^2^)	23.51 ± 2.9	24.4 ± 1.8	23.4 ± 2.2	NS
PRFT/BMI	0.2 ± 0.09	0.32 ± 0.01	0.13 ± 0.04	< 0.0001
Current smoker (%)	36.3	35.2	33.3	NS
Systolic pressure	123 ± 6.8	126.9 ± 7.3	122.0 ± 6.7	NS
HIV exposure				
Homosexual	42 (47.7%)	23 (46%)	19 (50%)	NS
Heterosexual	31 (35.2%)	17 (34%)	14 (36.8)	NS
IDU	15 (17%)	8 (16%)	7 (18.4%)	NS
HCV coinfection (%)	20.5	20	20.8	NS
CD4 (cells/μl)	534.3 ± 251.6	533.8 ± 271.2	535 ± 212	NS
HIVRNA load (mean log_10 _copies)	2.06 ± 1.2	2.1 ± 1.01	1.9 ± 1.4	NS
Duration of HAART (months)	75 ± 40.9	82.4 ± 35.9	70.4 ± 43.6	NS
Total cholesterol (mg/dl)	184.5 ± 44.7	190.3 ± 54.9	180.9 ± 37	NS
HDL cholesterol (mg/dl)	51.5 ± 15	52.6 ± 16.2	50.8 ± 14.4	NS
Triglycerides (mg/dl)	156.1 ± 94.7	177.3 ± 97.8	142.7 ± 91.3	NS
Glycemia (mg/dl)	94.3 ± 11.8	92.8 ± 6.9	86.7 ± 9.2	NS
2 NRTI + 1 NNRTI	41 (46.5%)	23 (46%)	18 (47.3%)	NS
2 NRTI + 1 PI	47 (53.4%)	27 (54%)	20 (52.6%)	NS

A, all HIV+ patients; B, HIV+ patients with visceral obesity; C, HIV+ patients without visceral obesity; BMI, body mass index; PRFT, perirenal fat thickness; IDU, intravenous drug users; HAART, highly active antiretroviral therapy; HDL, high density lipoprotein; NRTI, nucleoside reverse transcriptase; NNRTI, non-nucleoside reverse transcriptase; PI, protease inhibitor; NS, not significant. All data are expressed as mean ± standard deviation. A p-value of < 0.05 was considered to be statistically significant.

**Figure 3:**
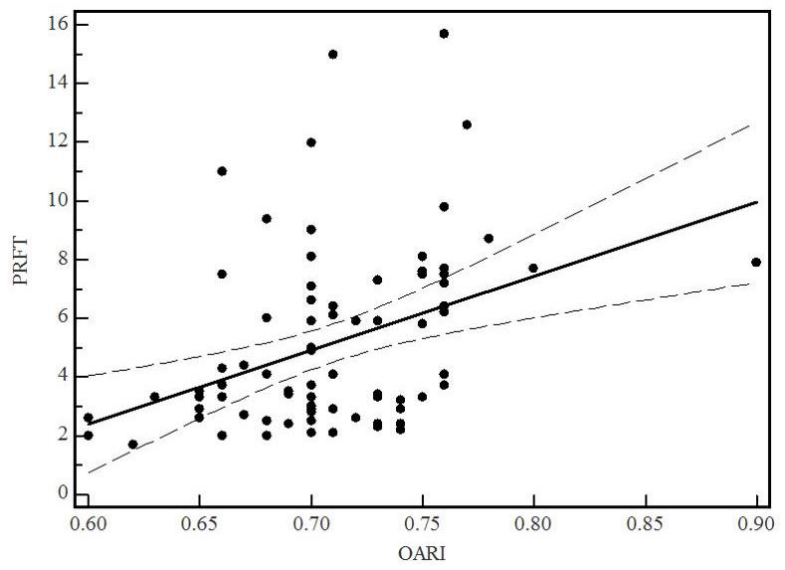
**Linear regression curve of relation between echographically measured PRFT and OARI in 88 HIV-1-infected patients**. Dotted line, 95% confidence interval; PRFT, perirenal fat thickness; OARI, ophthalmic artery resistance index

**Figure 4:**
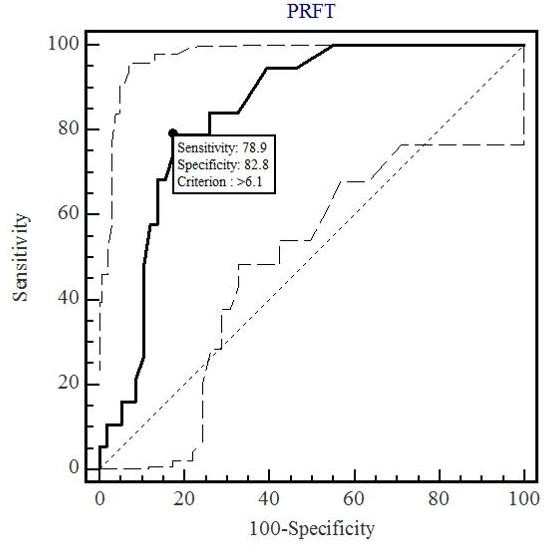
**Receiver operating characteristics curves of Perirenal Fat Thickness (PRFT) considering Ophthalmic Artery Resistance Index (OARI) > 0.74 as status variable**. The area under the curve was 0.84 (95% CI, 0.74 to 0.91; p < 0.001). CI, confidence interval

At baseline the mean of PRFT and OARI in HIV-1-infected patients with visceral obesity were considerably higher (8.1 ± 2.3 vs 3.2 ± 0.9 mm, p < 0.0001 and 0.73 ± 0.04 vs 0.69 ± 0.03, p < 0.001, respectively) than in patients without (Table 2). An OARI anomalous value (0.90 mm) in HIV 1+ patients (Group B) doesn't influence the mean values and the statistical analysis. For all 88 HIV-1-infected patients, US-measured PRFT correlated well with OARI (r, +0.52, p < 0.001) (Fig. 3). Receiver operating characteristic curves (ROC) indicated that the most discriminant PRFT value for predicting an OARI > 0.74 was 6.1 mm (sensitivity 78.9%, specificity 82.8%, AUC 0.84, p < 0.001) (Fig. 4). Using the average OARI as the dependent variable, total serum cholesterol (Odds Ratio 0.9, 95% CI 0.98 to 1.01), LDL (Odds Ratio 0.9, 95% CI 0.98 to 1.04) triglycerides (Odds Ratio 0.9, 95% CI 0.98 to 1.0), glycemia (Odds Ratio 0.9, 097 to 1.1), sex (Odds Ratio 1.0, 95% CI 0.96 to 1.05), blood pressure (Odds Ratio 0.8, 95% CI 0.98 to 1.07), age (Odds Ratio 1.13, 95% CI 1.04 to 1.23) and PRFT (Odds Ratio 1.44, 95% CI 1.12 to 1.85) were independent factors associated with OARI. No statistically significant correlations between PRFT and body mass index were found.

**Table 2:** Ultrasonographic PRFT and OARI measurement values in HIV-1 patients receiving HAART

Characteristic	A (n = 88)	B (n = 38)	C (n = 50)	p-value
PRFT	5.1 ± 3	8.1 ± 2.3	3.2 ± 0.9	< 0.0001*
OARI	0.70 ± 0.04	0.73 ± 0.04	0.69 ± 0.03	< 0.001**

A, all HIV+ patients; B, HIV+ patients with visceral obesity; C, HIV+ patients without visceral obesity; PRFT, perirenal fat thickness; OARI, ophthalmic artery resistance index. All data are expressed as mean ± standard deviation. * B vs C; ** B vs C. A p-value of < 0.05 was considered to be statistically significant.

## Discussion

Several reports have shown that HIV-1 patients have an increased risk of cardiovascular disease [19,20]. In addition, many epidemiological studies have shown increased visceral fat accumulation to be an independent risk factor for cardiovascular diseases such as coronary artery disease, stroke and hypertension [21-23].

Visceral obesity can be evaluated by different techniques, including CT [24-26], MRI [27-29] and ultrasonography [16,30-34]. However computed tomography (CT) and magnetic resonance (MRI) are expensive and the first method employs ionizing radiation, so they are not suitable for large-scale use. Sonography is simple, rapid, available, safe and is low-cost compared to CT and MRI.

In this study, the measurements were performed by a single operator, so it was not possible to assess the inter-operator variability.

The measurements made by two expert sonographers do not show significant differences [13], and another study confirms that a junior sonographer can be just as successful as a senior sonographer [7].

Kawasaki et al. [18] measured para- and perirenal ultrasound thickness and reported that it significantly correlated with fat thickness assessed by CT, according to previously published data [32]. Moreover, it was shown that scanning was simple and satisfactory images were obtained without interference of bowel gas.

For all these reasons, we used ultrasonographic methods to assess visceral fat distribution measuring the distance between the liver and the renal hilum of the right kidney.

PRFT is marker easily obtained during a routine ultrasound examination of the upper abdomen.

*This examination provides information on kidney and liver injuries as a result of HAART, on the infection itself or on comorbidity with other viruses (HBV, HCV)*.

*Furthermore, ultrasound assessment is an essential method for the study of clinical staging and monitoring of HIV patients*.

*The ophthalmic artery is the first major branch of the internal carotid artery, and changes in blood flow have provided new insights into various vascular disorders including carotid artery stenosis and metabolic disorders*.

*OARI offers particular advantages due to the absence of ultrasound obstacles and the vertical angle, which differs from the parallel-signaling of the carotid artery *[35].

Orbital circulation changes with varying degrees of carotid stenosis [36] were observed, with a significant relation between orbital velocity changes and carotid occlusive disease [37]. Studies on carotid artery stenosis have shown decreased blood flow velocity in the ophthalmic artery when the stenosis was greater than 70% [38], showing that measurement of orbital vessel velocity may be essential for evaluating the distal consequences of carotid artery stenosis [39].

Because carotid stenosis is an independent prediction of stroke and myocardial infarction in the general population [40-43], our data suggest that the rate of vascular events is likely to increase substantially in HIV patients receiving highly active antiretroviral therapy, who have been diagnosed with visceral obesity.

To our knowledge, this is the first study to compare visceral fat assessment and ophthalmic blood flow, in order to find a reproducible tool for early diagnosis of endothelial damage.

In conclusion, our data show that ultrasonographic measurement of PRFT could be considered a simple and objective parameter for early diagnosis of atherosclerosis. Furthermore, it allows identification of patients at increased risk of cardiovascular disease for which other clinical studies are useful.

### Clinical implications

From recent data, it appears that central fat distribution may be of more importance in endothelial damage than overweight alone, confirming previous studies showing that visceral fat accumulation itself played a role as a risk factor for atherosclerosis [44,45]. Ultrasound may represent an important step towards selecting patients at high risk for developing metabolic syndrome, allowing early intervention, and thus minimizing the impact of complications resulting from this condition [46].

Measurement of visceral obesity with ultrasound has the advantage of being non-invasive, easily accessible, economical and does not involve exposure to ionizing radiation. Limits to the use of VAT measurement are operator pressure, presence of bloating and poor accuracy for obese patients.

The determination of visceral fat by PRFT may be a more accurate method since perirenal fat contains only visceral adipose tissue while the single waist circumference or waist and hip ratio circumferences (WHR) cannot distinguish subcutaneous fat from intra-abdominal fat [7].

Furthermore, in HIV patients it has been observed that the determined value of ultrasound PRFT is particularly useful for diagnosing lipodystrophy. The available data indeed show that the thickness of the perirenal fat occurs earlier than accumulation of fat in other body areas [15].

## Abbreviations

HAART: highly active antiretroviral therapy; HIV: human immunodeficiency virus; PRFT: perirenal fat thickness; OARI: ophthalmic artery resistance index; IMT: intima-media thickness; HDL: high-density lipoprotein; NNRTI: Non-nucleoside reverse-transcriptase inhibitor; PI: protease inhibitor; VAT: visceral adipose tissue.

## Competing interests

The authors declare that they have no competing interests.

## Authors' contributions

PG wrote the manuscript; RC performed the ultrasound examinations; AZ and MG participated in writing the manuscript and performed the statistical analysis. All authors critically revised the manuscript.

All authors read and approved the final manuscript.
